# Perfect imaging, epsilon-near zero phenomena and waveguiding in the scope of nonlocal effects

**DOI:** 10.1038/srep02526

**Published:** 2013-08-28

**Authors:** C. David, N. A. Mortensen, J. Christensen

**Affiliations:** 1 Instituto de Química-Física Rocasolano, Consejo Superior de Investigaciones Científcas, 28006 Madrid, Spain; 2 DTU Fotonik, Department of Photonics Engineering, Technical University of Denmark, DK-2800 Kongens Lyngby, Denmark; 3 Center for Nanostructured Graphene (CNG), Technical University of Denmark, DK-2800 Kgs. Lyngby, Denmark; 4 Institute of Sensors, Signals and Electrotechnics (SENSE), University of Southern Denmark, DK-5230 Odense M, Denmark

## Abstract

Plasmons in metals can oscillate on a sub-wavelength length scale and this large-*k* response constitutes an inherent prerequisite for fascinating effects such as perfect imaging and intriguing wave phenomena associated with the epsilon-near-zero (ENZ) regime. While there is no upper cut-off within the local-response approximation (LRA) of the plasma polarization, nonlocal dynamics suppress response beyond *ω*/*v_F_*, where *v_F_* is the Fermi velocity of the electron gas. Nonlocal response has previously been found to pose limitations to field-enhancement phenomena. Accounting for nonlocal hydrodynamic response, we show that perfect imaging is surprisingly only marginally affected by nonlocal properties of a metal slab, even for a deep subwavelength case and an extremely thin film. Similarly, for the ENZ response we find no indications of nonlocal response jeopardizing the basic behaviors anticipated from the LRA. Finally, our study of waveguiding of gap plasmons even shows a positive nonlocal influence on the propagation length.

Plasmonics has provided us with a number of fascinating optical phenomena and taught us ways to squeeze light down to the nanometer-scale. Surface plasmons are guided electromagnetic (EM) waves confined at the interface between a metal and a dielectric, which originate from collective interaction of light and free conducting electrons. Current research, with emphasis on applied aspects, focuses on the design and experimental characterization of sensors^1^,^2^, communication devices, such as compact optical switches^3^, waveguides^4^ and detectors all on the nanoscale. One of many challenges within this framework is to provide integrated building blocks in order to facilitate high-bandwidth and long-range plasmon propagation for many intriguing engineering application. Beyond these technical complexities, there are other more fundamental ingredients in plasmonic structures playing an important role, in need to be regarded, when seeking for maximal field enhancements, super focusing and improving the figures of merits within these components. Apart from resistive losses of metals, the dominant role played by quantum effects and, in particular, intrinsic nonlocality in the dielectric response are known to be limiting factors when aiming at highest plasmonic robustness for the full control of light^5^,^6^,^7^.

In this work, we investigate planar plasmonic systems when taking into account the effects of nonlocality. Our aim is to look at basic building blocks and their striking functionalities and we wish to demonstrate how their properties can be altered implementing the nonlocal nature within the dielectric response. Nonlocal effects arise from the quantum nature of the free electrons and their interactions in metals^8^,^9^,^10^,^11^,^12^. Including the spill-out of valence electrons, narrow gaps or molecules in the proximity of a metallic surface allow for complex interactions with the environment. Recent experiments explore this frontier^13^,^14^,^15^,^16^,^17^ that has come into reach for modern nano-fabrication methods. The hydrodynamic approach^7^,^18^,^19^,^20^,^21^,^22^,^23^,^24^,^25^,^26^ and other semi-classical theories^6^,^27^,^28^,^29^,^30^ have proven important for analyzing the observed effects. In the hydrodynamic model, 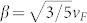 quantifies the strength of the nonlocal response associated with hydrodynamic pressure due to electron-electron interactions, where *v_F_* is the material dependent Fermi velocity. For larger systems, edge effects due to the electron spill-out play a minor role compared to nonlocal bulk effects that are subject to the uniform electron distribution inside the metal structure.

Metal slabs provide a rather simple, yet rich in view of possible functionalities, structure to test various plasmonic properties in the scope of nonlocality. In the context of the Veselago lens^31^, Pendry showed that a thin silver layer treated within the extreme near-field limit and radiated with p-polarized light at a frequency when 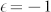 could function as a “poor-man's superlens” with seemingly unlimited spatial resolution^32^. A sub-diffraction limited source can thus be perfectly imaged at the far-side of the slab by its propagative wave components, but most importantly, also by the amplified evanescent component hosting all important near-field information^32^,^33^. Nonlocal response is generally associated with a long-wavevector cutoff^7^ which could have detrimental consequences for perfect imaging. We show with the hydrodynamic model that perfect imaging is only marginally affected by nonlocal properties of a metal slab, even for a deep subwavelength case and an extremely thin film. This supports the conclusion of previous work where the nonlocal dynamics was accounted for by hydrodynamic models^34^ and more elaborate density-functional theory within the time-dependent local-density approximation^35^ confirming the robustness of this superlens concept. In a related context, when radiating a silver slab at its plasma frequency, the electric response is suppressed and the permittivity zero. It is known that a material slab with epsilon-near-zero (ENZ) will give rise to tunneling and super-coupling related phenomena^36^,^37^,^38^,^39^. Operating at ENZ will emit the radiated waves of a point source through a slab in a directive pattern due to zero phase change within such materials. Our aim is to study this phenomena at the nanoscale by taking into consideration nonlocal effects. Finally, these effects will be also considered in metal-insulator-metal (MIM) waveguides that are known for supporting long-range propagating surface plasmons^40^,^41^,^42^. Interestingly, we do find propagation lengths in these MIM waveguides to be increased due to the nonlocal properties of the metal regions.

We provide a tutorial and entirely analytical insight into planar plasmonic structures with illustrative metamaterial-inspired examples. In this framework we show that the hydrodynamic model representing the nonlocal nature of the optical response can be cast into a simple wave equation. Solving the respective boundary value problems, we arrive at the analytical nonlocal analogue of Fresnel's coeffcients for abrupt interfaces, i. e. a uniform electron distribution inside the metal, which neglects the effect of the electron spill-out. This results in additional longitudinal modes not present in the local approximation. Differences in the performance of plasmonic devices compared to the local approximation are discussed on the basis of this analytic study.

## Results

### Nonlocal Fresnel coefficients

Fresnel's optical coefficients describe the behaviour of EM waves crossing a planar interface between media of differing refractive indices. Our aim is the derivation of these coefficients in single slab environments consisting of metal films with Drude dielectric function 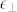 surrounded by dielectric material with permittivty 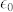 and vice-versa including nonlocal properties of the free electron gas in the metal regions. The electric field in nonlocal media consists of transversal and longitudinal components in contrast to the local approximation, where only transversal waves are reflected and transmitted at a metal to dielectric interface. This leads to the wave numbers 
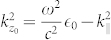
, 
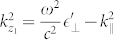
 and *q* associated to light propagation in the dielectric and to transversal and longitudinal waves in the metal, respectively. Note that we consider p-polarized light that supports surface plasmons. The classical Fresnel equations do not change in case of s-polarization, assuming non-magnetic media with permeability *μ* = 1.

We solve for the coefficients of reflection *r* and transmission *t* of a single metal-dielectric interface by evaluating the retarded Maxwell's equations together with the linearized hydrodynamic equation using appropriate boundary conditions^21^,^43^. Details on this procedure are given in the methods section. Note, that the incident wave is always considered to be transversal. However, on entering a nonlocal metal both transversal and longitudinal polarizations are excited. Considering multiple reflections inside a metal slab, the case of a transversal wave incident on a metal-to-dielectric interface has to be regarded. This results in three sets of optical coefficients discussed next. At a dielectric-to-metal interface the following transversal (index *p*) and longitudinal (index *l*) modes are found for p-polarization 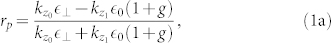

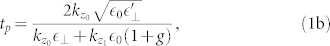

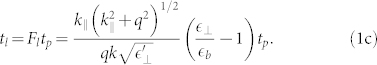
Likewise, for a transversal wave incident on a metal-to-dielectric interface we have 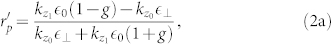

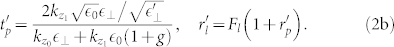


Finally, for the case of a longitudinal wave incident on a metal-to-dielectric interface we write 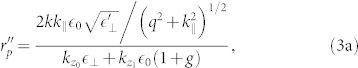

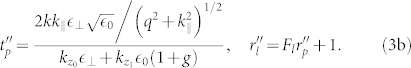


Without loss of generality, these expressions also include intrinsic material losses via the complex permittivity of the materials. The overall form of the transversal modes is similar to the local Fresnel coefficients, but the resonance structure now depends on the parallel momentum via the term 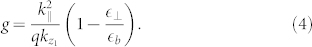
In the local limit (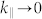), the usual optical coefficients are recovered and the additional longitudinal contributions, not present in the local case, vanish. Note that the strength of the nonlocal response *β* is introduced in these equations via the wave number of the longitudinal wave 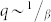. For a metal slab or an MIM structure, we need to consider multiple reflections in the inner layer. In contrast to the local approximation, additional contributions from scattered longitudinal waves are found and we therefore need to sum all components to arrive at the overall expressions for the transmittance and reflectance |*T*|^2^ = |*T*_trans_|^2^ + |*T*_long_|^2^ and |*R*|^2^ = |*R*_trans_|^2^ + |*R*_long_|^2^.

### Image of a subwavelength source

Pendry predicted that negative refraction makes a perfect lens^32^. While initially this concept faced some criticism it was quickly realized and experimentally verified that perfect imaging is possible although limited due to intrinsic material losses^32^,^44^. It is our aim to test the feasibility of this perfect lens in the scope of nonlocality. The usual way a very small object emits light is by propagating waves which can be seen under a conventional microscope. We are, however, more interested in the finer details of the near-field that are difficult to detect. Using a perfect lens, we can amplify the evanescent near-field waves and principally being able to refocus the small object onto the image plane in the far-side of the flat lens. The poor-man's superlens, as provided by a good conductor below the plasma frequency 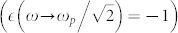, decouples magnetic contributions from electrical ones in thin films. This allows for using the derived Fresnel coefficients for the case of p-polarized light. These nonlocal expressions reveal a complex resonance structure originating not only from the resonance frequency mismatch induced by the additional term *g* in the single surface optical coefficients, see Eq. (4), but also from additional contributions given by longitudinal waves not present in the local approximation 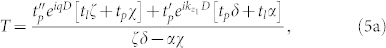
with the coefficients 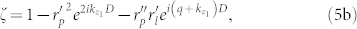

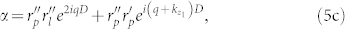

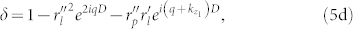

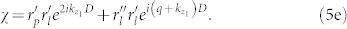


**Figure 1:**
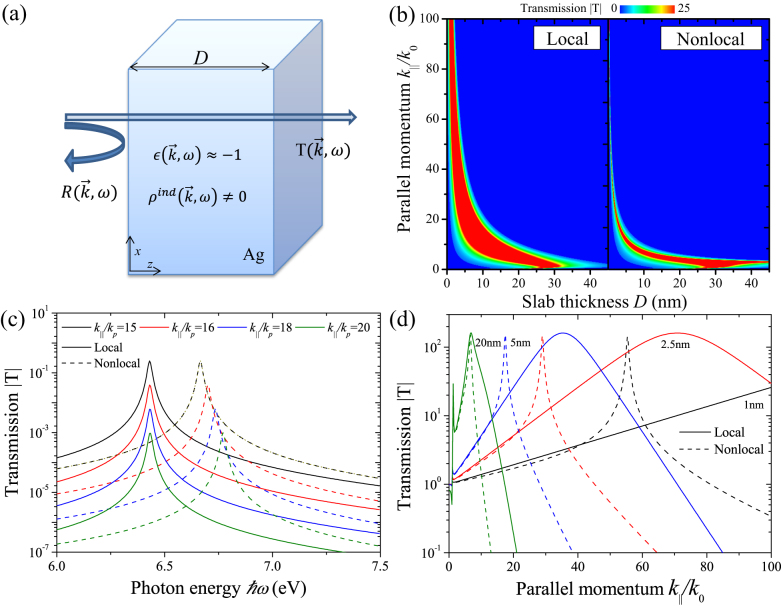
Optical properties of a silver slab at 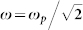 in the local approximation and with nonlocal response. (a) Illustration of the setup under consideration using a silver slab with Drude response using *ω_p_* = 9.1 eV and *γ* = 0.02 eV. (b) Transmission |*T*| as a function of parallel momentum 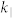 and slab thickness *D* including realistic damping *γ*. (c) At specific parallel momenta 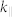 we plot |*T*| as a function of photon energy ℏ*ω* for *D* = 10 nm. The values chosen are normalized by 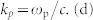. (d) Here the frequency is again locked at 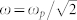 but for various slab thicknesses, as indicated, we plot 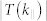.

**Figure 2:**
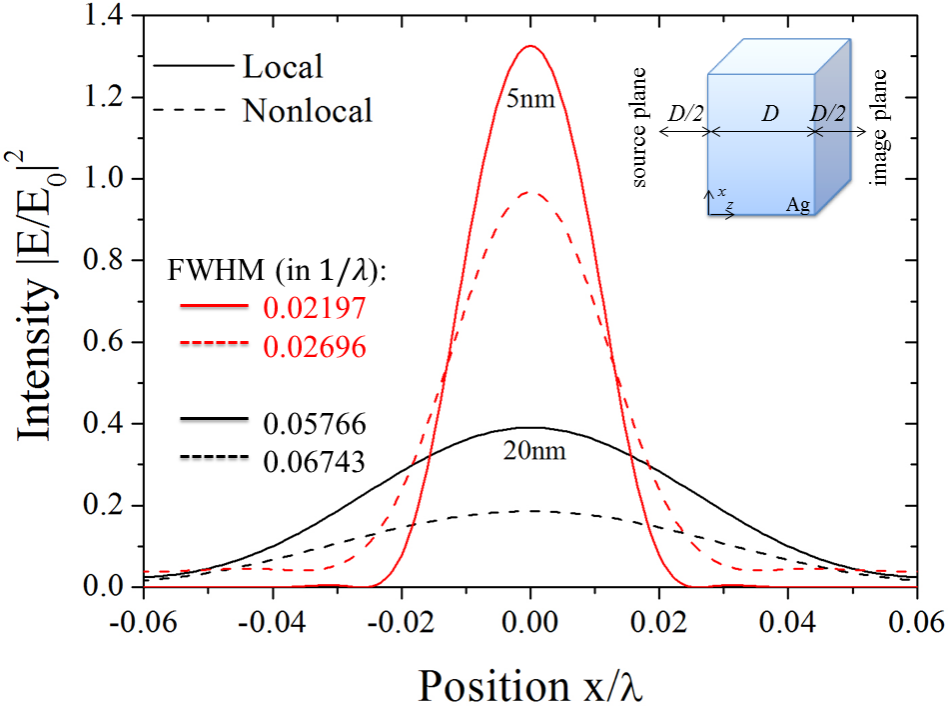
Perfect imaging of a deep subwavelength source by nonlocal metal slabs. Normalized intensity profile at the image-plane using two different thicknesses, *D* = 5 nm and 20 nm. A hat-function of linewidth *λ*/30, constitutes a subwavelength light emitting source and is placed at a distance *D*/2 in front of the lens with thickness *D*. The image plane locates at *D*/2 behind the slab. The FWHM values are given for each case to demonstrate the small change of imaging quality.

Note that in the local limit *α*, *χ* → 0 and *δ* → 1, whereby *ζ* becomes the known denominator for the optical coefficients of a local slab of thickness *D*. Fig. 1(b) shows the transmission Eq. (5a) versus slab thickness and parallel momentum (normalized by *k*_0_ = *ω*/*c*) at imaging conditions, i. e. 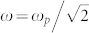, including intrinsic resistive losses. We predict a reduction in the momentum bandwidth for the presented nonlocal case, which is a direct consequence of additional loss channels introduced by the augmented sensitivity with parallel momentum via Eq. (4). The coupling to the longitudinal modes, not present in the local approximation, results in sharpened resonances shifted towards lower parallel momenta as seen in Fig. 1(d). For increasing slab thicknesses the nonlocal results converge to the local limit. In all cases however, both for the local and nonlocal examples, as expected we find transmissions exceeding unity due to the amplification of evanescent waves. Spectrally, as illustrated in Fig. 1(c), we reconfirm that optical modes become dependent on 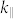 in nonlocal theories both for their resonance position and width. The resultant blueshift leads to a reduction in the transmission for increasing spatial dispersion. In other words, a local metal slab shows resonances independent with parallel momentum (for high enough 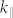), while in the nonlocal case a frequency mismatch is observed that depends crucially on the parallel momentum. From these results we expect consequences for perfect imaging. The performance of nonlocal lenses under perfect imaging conditions has been investigated previously^5^,^34^,^35^. As rendered in Fig. 2 we use a deep subwavelength source of width *λ*/30 and investigate the imaging properties of extremely thin metal films, *D* = 5 nm and 20 nm. Although the foregoing study reveals a reduction in 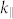 bandwidth, we do not find severe deterioration in the imaging quality, since the emitted near-field of the quasi point-source does not exceed the available 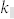 spectrum. Thus, the difference in the bandwidth does play a minor role. This is further confirmed by the values of the FWHM calculated for each case and indicated in Fig. 2. Therefore, we conclude, that nonlocality is not a considerable limiting factor for lensing applications, given only minor differences in the quality of the image as illustrated by the intensity profiles in Fig. 2.

### Nonlocal epsilon-near-zero metal slabs

**Figure 3:**
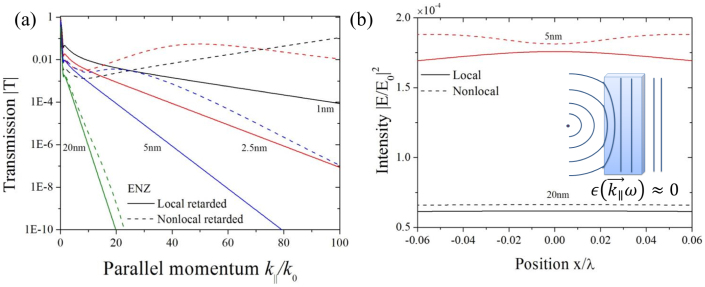
Local and nonlocal metal slabs at ENZ condition *ω* ≡ *ω_p_*. (a) We plot the transmission |*T*| as a function of 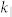 for various slab thicknesses *D* for a silver slab in Drude response with *ω_p_* = 9.1 eV and *γ* = 0.02 eV. (b) At *D*/2 in the far-side of the ENZ slab, intensity profiles for slabs of *D* = 5 nm and 20 nm are plotted for a light emitting source placed at distance *D*/2 in front of the slab.

The effective wavelength of an electric field in a material at a frequency with zero permittivity (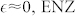) can become very large and provides very small phase variations over physically long dinstances. This enables the efficient transport of electromagnetic energy through subwavelength channels and yields enhanced radiation directivity in media operating at the ENZ condition, see inset in Fig. 3(b). These exciting optical properties can be artificially created by metamaterials to work at any desirable frequency. However, metal films exhibit the same property at their plasma frequency *ω_p_* allowing to study ENZ phenomena which rely on the intrinsic behaviour of a metallic medium.

We apply the above equations (5) for nonlocal thin metal films at a frequency *ω* = *ω*_p_, which leads to 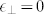 in the local case. For a local metal thin film, full transmission at this frequency is possible only for radiation at normal incidence^38^ 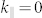, see Fig. 3(a). While this also is the case for the nonlocal situation, an increased transmission with parallel momentum is found due to additional longitudinal modes. The frequency mismatch of the nonlocal case is increasing with the parallel momentum and leads to an actual mismatch of the ENZ condition. An increase of several orders of magnitude in the transmission is observed, giving rise to almost fully transparent thin nonlocal metal films for higher parallel momenta. Since the transmission shows sensitivity to spatial dispersion we expect that directive radiation will be influenced by the nonlocal contributions. Light emitted by a point source, placed at the nearest vicinity (with distance *D*/2 from the first interface) of an ENZ material slab, propagates with uniform phase distribution inside the material. Providing sufficient material thickness, the wave remains directive upon emerging the broadside of the slab^38^. This is shown in Fig. 3(b) with the intensity profile captured at *D*/2 in the far-side for the case of *D* = 20 nm. In the extreme limit comprising a slab of *D* = 5 nm thickness, as already depicted in Fig. 3(a), the uniformity of the phase is broken leading to an increase of the transmitted energy. However, the slab is already too thin to provide full phase-alignment when emerging the region of ENZ, hence for realistically designed systems working as super-couplers and directive antennas, minor influence is to be expected within the scope of nonlocality.

### Waveguiding properties

Apart from the optical properties of bare metal slabs, we also study the implications of nonlocal optical response in metal-insulator-metal (MIM) waveguides. Surface plasmon based MIM waveguides are used to guide light in volumes far beneath the diffraction limit, which is enabled by a strong confinement of the surface plasmons to the metallic surfaces. This is in contrast to single metal-insulator interfaces where high propagation losses are observed.

**Figure 4:**
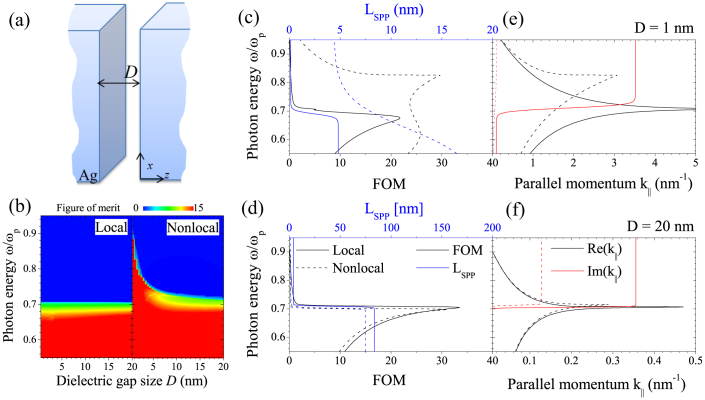
Waveguiding properties of MIM structures including nonlocal optical response. (a) Illustration of the silver MIM structure under consideration. (b) The figure of merit (FOM) for local and nonlocal MIM structures as a function of *ω* (normalized to the plasma frequency *ω*_p_) and the dielectric gap size *D*. (c), (d) FOM (black curves) and SPP propagation length *L*_SPP_ (blue) in the local approximation (solid) and including nonlocality (dashed). (e),(f) Photonic band structure of the MIM waveguides comparing the local and nonlocal theory. Simulations are conducted with gap size *D* = 1 nm in (c) and (e) and *D* = 20 nm in (d) and (f).

In the present study we examine the quality and properties of waveguiding in MIM structures in the presence of additional longitudinal modes originating from nonlocal properties of the metal regions. In the same theoretical framework as presented earlier for the bare metal slab, we derive reflection coefficients comprising transversal and longitudinal optical propagation for the MIM configuration, see Fig. 4(a), assuming an incoming transversal wave, 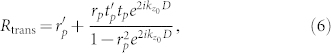

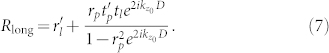


By inspecting the poles of the denominator in the reflection coefficient, we are able to calculate the band structure such as the linewidth of the resonances. In other words, for a given frequency we can evaluate the real and imaginary part of the parallel wave vector in order to express the figure of merit (FOM) and the propagation length of the surface plasmons (*L*_SPP_) 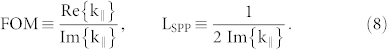
Results are depicted in Fig. 4 that show a remarkable benefit from the nonlocal properties of the metal regions in comparison to the local case^42^. We begin by calculating the FOM as a function of frequency and dielectric gap size *D* as presented in Fig. 4(b). The local approximation shows an abrupt transition from high to low FOM right at the SPP resonance^42^. In the nonlocal case this FOM edge is blueshifted for gap separations *D* ≤ 5 nm and leading to a remarkably high FOM for small gap sizes beyond the plasmon resonance. Next, we consider two representative dielectric gap sizes from Fig. 4(b), *D* = 1 nm and *D* = 20 nm. Again, we use the expressions for the propagation length *L*_SPP_ and the FOM given by Eq. (8) and plot their dependence with frequency for the two specific configurations, see Fig. 4(c) and 4(d). While the case for *D* = 20 nm shows minimal differences, Fig. 4(c) for *D* = 1 nm again highlights the improved quality such as enhanced propagation lengths when including a nonlocal dielectric response. In order to trace back the origin for this improvement we plot the complex band diagrams as illustrated in Fig. 4(e) and 4(f). In the local approximation the plasmon propagation length, due to an almost constant imaginary part of 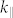, remains unaltered for the two specific values of *D*. In the nonlocal picture however, 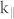 is severely reduced which means that MIM structures sustain resonances of high lifetime and consequently give rise to impressively large FOMs. As expected, this prediction is most notable for the case with *D* = 1 nm. For the larger dielectric gap, only slight differences are found in the bandstructure between the local and nonlocal case. With decreasing size, we not only observe a blueshift of the resonance, see Fig. 4(e), but the aforementioned enhanced FOM is also explained by a reduction in the linewidth of the mode. Interestingly, the predicted plasmon propagation length within a 1 nm dielectric gap is itself only a fraction of a nanometer, but above the plasmon resonance (*ω*/*ω_p_* > 0.7) it is orders of magnitude larger than in the local approximation.

## Discussion

Within an analytical framework we have inspected plasmonic building blocks and their use for superlenses and ENZ applications such as waveguiding. We have derived exact expressions for the nonlocal Fresnel coefficients through a semiclassical hydrodynamic approach. Through them, we demonstrated that nonlocality does not necessarily spoil the optical phenomena found for surface plasmon related phenomena comprising strong dependence with spatial dispersion.

We can conclude that negative refraction, also in the quasi-static approximation, taking into account nonlocal contributions does not put the prospects of the pefect lens at stake. The effects of nonlocality are only marginally influencing this concept. The same applies to ENZ applications, but the propagation length and figure of merits in MIM structures are remarkably increased, altogether paving a way for novel plasmonic applications even in the scope of nonlocal interactions.

## Methods

### Hydrodynamic framework

In the hydrodynamic model we treat the dynamics of the free electron gas in terms of a change in the induced charge and current density, and the core-polarization within the metal with dielectric background 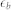 separately^25^. We introduce the transversal dielectric function 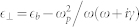 of a metal with the free electron contribution defined via its characteristic plasma frequency *ω_p_* and the inverse lifetime of plasmonic excitations *γ*. Throughout this article, we study silver slabs in Drude response and waveguides with *ω_p_* = 9.1 eV, *γ* = 0.02 eV and 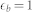.

In the linearized hydrodynamic equation 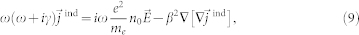
where the parameter 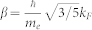 introduces nonlocal effects. This is defining the pressure of a fully degenerated electron gas subjected to Coulomb interaction^45^,^46^ which accounts partly for the quantum nature of the conduction band electrons. The coupling between the light wave and the electron current density is given by 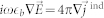. This leads to the wave equation 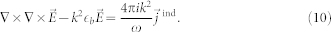
Combining eqs. (9) and (10), we can rewrite the wave equation into^43^

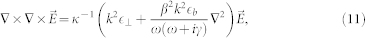
where the vector identity 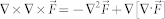 was used. Noting that the small parameter *κ* is approximately 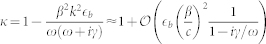, we neglect the inverse term on the right hand side and arrive at a generic wave equation that is independent of the current density and is very suitable for implementation in any numerical framework enabling the study of arbitrary structures^43^. In this work, however, we solve these equations analytically for planar metal-dielectric interfaces.

The inhomogeneous solution of the electric field determines the longitudinal modes. Those are unaltered by the introduced approximation. Note, that the transversal part of the electric field inside the nonlocal metal is governed by 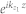 and the longitudinal solutions propagate with *e^iqz^* with 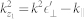 and 
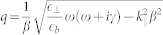
. With the generic wave equation derived above 
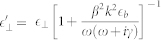
. The additional factor appearing here is again small and leads for all investigated cases to the same results as the full wave equation result.

### Boundary conditions and longitudinal solution

To derive the nonlocal Fresnel coefficients at an interface with surface normal 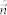, we need to evaluate the solutions of the above equations with suitable boundary conditions. The hydrodynamic equation requires an additional boundary condition, which demands the continuity of the normal component of the current density. This leads to a set of three equations for a metal-dielectric interface given by 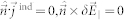 and the continuity of 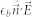. Since surface plasmons are supported by incident p-polarization (TM) waves, we restrict ourselves to this case.

After some algebra we rewrite the wave equation to 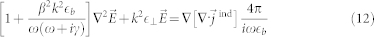
and find the solution for the inhomogeneous (longitudinal) part to read 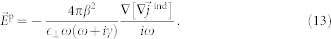
Finally, one can solve a set of equations given by the above boundary conditions. Furthermore, depending on the wave nature of the incoming wave (transversal or longitudinal) such as the composition of the interface (metal to dielectric or vice-versa) we arrive at the optical coefficients presented with eqs. (1a)–(3b).

## Author Contributions

J.C. and N.A.M. conceived the idea and lead the project. C.D. and N.A.M. developed the numerical model. C.D. performed all calculations, created the figures and wrote the manuscript. All authors participated in the revision.
